# Safety profile of *Dioscorea hispida* tuber extract: a combined acute toxicity and genotoxicity study

**DOI:** 10.3389/fphar.2025.1666101

**Published:** 2025-09-08

**Authors:** Hussin Muhammad, Nik Aina Syazana Nik Zainuddin, Nur Liana Md Nasir, Siti Soleha Ab Dullah, Elda Nurafnie Ibnu Rasid, Mei Siu Lau, Mohd Rahimi Ashraf Abd Rahman, Chin Long Poo, Siti Khadijah Mustapha Kamal, Norizah Awang

**Affiliations:** Toxicology and Pharmacology Unit, Herbal Medicine Research Centre, Institute for Medical Research, National Institutes of Health, Ministry of Health, Setia Alam, Selangor, Malaysia

**Keywords:** *Dioscorea hispida*, genotoxicity, micronucleus, acute toxicity, mutagenicity

## Abstract

**Introduction:**

*Dioscorea hispida* has been widely known for consumption as food due to its medicinal properties. The present study evaluated the safety of *D. hispida* tuber aqueous extract through single-dose toxicity and genotoxicity assessments.

**Methods:**

An acute oral toxicity study was conducted with single oral doses of 5, 50, 300, and 2000 mg/kg body weight *D. hispida* aqueous extract in respective groups. The bacterial reverse mutation test was performed on *Salmonella typhimurium* strains TA100, TA1535, TA98, TA1537, and *Escherichia coli* strain WP2uvrA using the pre-incubation method, both in the presence and absence of an exogenous metabolic activation system (S9). An *in vitro* micronucleus assay was carried out on V79B cells, a fibroblast-like cells. The cells were treated with *D. hispida* aqueous extract at concentrations of 1.25, 2.5, and 5 mg/ml, along with positive or negative controls (distilled water). The cells were stained with acridine orange, observed under a fluorescence microscope, and scored for micronuclei.

**Results:**

Our findings showed the administration of *D. hispida* aqueous extract did not cause any adverse effects up to 2000 mg/kg body weight. Additionally, *D. hispida* aqueous extract did not induce mutagenicity in tested *Salmonella* and *E. coli* tester strains and did not increase revertant colonies at concentrations up to 5000 μg/plate. No significant changes were observed in the number of micronucleated cells compared to the untreated group.

**Discussion:**

*D. hispida* aqueous extract is not toxic in both *in vivo* acute toxicity and *in vitro* genotoxicity studies. However, further investigations are needed for preclinical studies on repeated administration and *in vivo* genotoxicity assays.

## 1 Introduction

Nature offers a wide array of foods with medicinal and nutritional value, including leaves, flowers, roots, tubers, fruits, and nuts (Chandrasekara and Josheph Kumar, 2016). Among these, roots and tubers are crucial in combating food scarcity, particularly in developing countries, yet there is insufficient documented scientific information on these crops (Kumar et al., 2012). Medicinal plants with bioactive compounds have pharmacological potential in drug discovery for pharmaceutical applications, necessitating both preclinical and clinical studies to evaluate their efficacy and safety for human use.

Over 630 *Dioscorea* species have been identified in subtropical and tropical regions worldwide (Dutta, 2015). The tubers of various *Dioscorea* species are traditionally used to treat a range of ailments in single or multiple formulations. *D. hispida*, known locally as ‘ubi gadung’ in Malaysia, is a poisonous tuber plant containing the alkaloid dioscorine. Research indicates that dioscorine extracted from *D. hispida* tubers can cause nausea, vomiting, dizziness, and sleepiness in humans as well as potentially fatal nervous system paralysis (Reddy, 2015; Liu et al., 2007). Traditionally, *D. hispida* tubers are only edible after undergoing detoxification in flowing water for a week to remove the dioscorine (Sasiwatpaisit et al., 2014; Kamaruddin et al., 2020; Azman et al., 2015).

*D. hispida* contains bioactive compounds such as diosgenin (steroidal saponins), dioscorine, and water-soluble polysaccharides (WSP) (Estiasih et al., 2014). Steroidal saponins have been reported to exhibit pharmacological activities, including anthelmintic, antioxidant, analgesic, anti-inflammatory, hypocholesterolemic, and anti-tumor effects (Avula et al., 2014; Nashriyah et al., 2012). In traditional Thai medicine, *D. hispida* dried tuber, known as “Thoraneesanthakhat,” is used as a crude drug to treat constipation. WSP has demonstrated antioxidant activity protecting DNA (Wang et al., 2011), immunostimulant (Liu et al., 2008), and antihypertensive effects due to its binding to dioscorine, as well as hypoglycemic activity (Estiasih T, 2011). *D. hispida* tubers also exhibit antimicrobial activity against bacteria and fungi such as *Escherichia coli*, *Staphylococcus aureus*, *Salmonella typhimurium*, and *Saccharomyces cerevisiae* (Azman et al., 2015; Cui et al., 2014). Additionally, the zebrafish embryo toxicity test indicated tolerance to *D. hispida* starch at acceptable concentrations, suggesting its suitability as an antimicrobial agent with minimal negative impact on vertebrates (Azman et al., 2015).

Despite the various pharmacological activities of *D. hispida*, pre-clinical safety data on its general toxicity and potential genotoxicity remain limited. Therefore, the present study was conducted to establish the safety profile of *D. hispida* tuber aqueous extract by assessing acute oral toxicity and *in vitro* genotoxicity.

## 2 Materials and methods

### 2.1 Chemicals

The chemicals used in this study were as follows: acridine mutagen ICR-191 (Sigma-Aldrich Chemical Co., Milwaukee, WI, United States), 2-Nitrofluorene (97.9% pure, Sigma-Aldrich Chemical Co.), 2-Aminoanthracene (97.5% pure, Sigma-Aldrich Chemical Co.), furylfuramide (99.7% pure, Wako Pure Chemical Industries Ltd., Osaka, Japan), sodium azide (99.7% pure, Merck, Darmstadt, Germany), dimethyl sulfoxide (DMSO, 99.99% pure, Sigma-Aldrich Chemical Co., St. Louis, MO, United States), the positive control Lot D00151128 from Calbiochem (without S9 metabolic activation), cyclophosphamide monohydrate 2886953 from Merck (with S9 metabolic activation), the lyophilized rat liver S9 mixture (Molecular Toxicology Inc., Boone, NC, United States), all strains), bacteria strains were obtained from Molecular Toxicology Inc., (Boone, NC United States), Dulbecco’s Modified Eagle’s Medium (Gibco, United States), fetal bovine serum (JR Scientific, United States) and antibiotic/antimycotic solution (Gibco, United States).

### 2.2 Plant material and extract preparation

Fresh *D*. *hispida* var daemona from the family of *Dioscoreaceae* tubers, aged around 2–3 years, were harvested from Kg Bukit Bakar, Machang, Kelantan based on visual maturity markers and authenticated by a botanist and the voucher specimen was deposited at the Forest Research Institute of Malaysia (FRIM) (Voucher No. SBID:0012/08). The rhizomes were peeled, cut into smaller pieces, washed and oven-dried at 50–60 °C for 2 days. The dried rhizomes were then ground into fine powder. This powder was subjected to 100% aqueous extraction, followed by centrifugation at 3,500 rpm for 10 min. The resulting supernatant was spray-dried to produce a fine powder which was stored at 4 °C for future use. The aqueous extract was sent to FRIM for qualitative phytochemical analysis. From our previous study (Muhammad et al., 2019; Muhammad et al., 2022), the dried extract (10 mg/mL) was filtered through a 0.22 μm PTFE membrane and analyzed using UHPLC-ESI-MS equipped with a WATERS C18 column and a gradient mobile phase of water and acetonitrile (both with 0.1% formic acid). Total polyphenol content was quantified using the Folin-Ciocalteu assay, which measures oxidized phenols via blue color intensity. The aqueous extract of *D*. *hispida* contained a total phenolic content of 243 ± 2.1 mg gallic acid equivalent (GAE) per 100 g. UHPLC-ESI-MS analysis revealed the presence of four steroidal saponins, which were tentatively identified based on their retention times, accurate molecular ions (m/z), molecular formulas, and comparison with reference standards and existing literature. Based on our preliminary data also, the LC-MS ESI chromatogram and spectral data showed four key phytochemical compounds were identified in the aqueous extract of *D. hispida.* These include protocatechuic acid (Rt: 0.80 min, m/z 153.02), phloretic acid (Rt: 2.54 min, m/z 165.05), 4-hydroxybenzoic acid (Rt: 3.69 min, m/z 137.02), and dioscin (Rt: 11.49 min, m/z 867.47). Each compound showed low mass error (1.7–7.1 ppm), indicating accurate identification.

#### 2.2.1 Alkaloids test

Powder from *D. hispida* aqueous extract was macerated in chloroform followed by the addition of ammoniacal chloroform. The extract was then treated with 10% sulphuric acid and tested with Mayer’s reagent. The formation of white precipitates indicates the presence of alkaloids.

#### 2.2.2 Saponins test

Methanol extract of *D. hispida* aqueous extract was mixed with distilled water in a test tube. The presence of saponins was indicated by the formation of stable froth for at least 15 min.

#### 2.2.3 Flavonoids test

The chloroform extract of *D. hispida* aqueous extract powder was dissolved in ether and shaken in a 10% ammonia solution. The formation of yellow colour in the ammonia layer indicates the presence of flavonoids.

#### 2.2.4 Tannins and polyphenolic compounds test

The methanolic extract of *D. hispida* aqueous extract was mixed with 1% ferric chloride solution blue black colour and brownish-green colour formed indicating the presence of hydrolysable tannins and condensed tannins respectively.

#### 2.2.5 Triterpenes/steroids test

Lieberman-Buchard reagent was used to test the chloroform extract of *D. hispida* aqueous extract and the formation of reddish and greenish colour indicate the presence of triterpene and steroids respectively.

### 2.3 Animal

Eight female Sprague Dawley (SD) rats, aged 6–8 weeks and weighing 180–200 g, were obtained from the Laboratory Animal Research Unit at the Specialized Resource Centre, Institute for Medical Research (IMR). The rats were housed individually in ventilated cages under standard environmental conditions (temperature: 22 °C ± 3 °C; humidity: 50%–60%) with a 12-h light-dark cycle. They were provided with a standard pellet diet and water *ad libitum.*

#### 2.3.1 Study design

The study followed OECD testing guidelines 420 for chemicals, using a fixed-dose procedure with minor modifications. A sighting study was first conducted to determine the appropriate starting dose for the main study. In this phase, one rat in each sighting group received a single oral dose of 5, 50, 300, and 2,000 mg/kg body weight of DHAE, dissolved in distilled water. Since no mortality occurred at the highest dose (2,000 mg/kg), this dose was selected for the main study with an additional four rats. Rats were monitored for signs of toxicity, including changes in skin, fur, eyes, mucous membranes, respiratory and circulatory effects, autonomic and central nervous system responses, as well as somatomotor activity, behaviour, tremors, convulsions, salivation, diarrhoea, lethargy, sleep, and coma at intervals of 0.5, 1, 2, 3, and 4 h, and then twice daily. Body weight was measured weekly before *D. hispida* aqueous extract administration for 14 days. Food and water intake were also recorded weekly. On day 15th, the rats were anesthetized with isoflurane, and blood samples were collected for biochemical analysis. Vital organs including the liver, heart, lungs, spleen, stomach, gastrointestinal tract, kidneys, ovaries, and adrenals were collected and weighed for further analysis.

#### 2.3.2 Biochemical analysis

Serum biochemical parameters were assessed using the Siemens Dimension EXL with LM Integrated Chemistry System (Siemens Healthcare Diagnostics, United States). The parameters measured included albumin, albumin/globulin (A/G) ratio, alkaline phosphatase (ALP), alanine aminotransferase (ALT), amylase, aspartate aminotransferase (AST), blood urea nitrogen (BUN), calcium, cholesterol, glucose, total bilirubin, total protein, and globulin. Blood collection was performed by trained laboratory personnel under the supervision of a licensed veterinarian. All biochemical analyses were conducted using validated standard clinical chemistry methods. Albumin was quantified using the bromocresol purple method, while total protein was measured via the modified biuret method. ALT was analyzed based on the modified International Federation of Clinical Chemistry (IFCC) method, and both AST and ALP activities were determined using kinetic assays. BUN was measured enzymatically, calcium using the ortho-cresolphthalein complexone (OCPC) method, cholesterol via an enzymatic cholesterol oxidase method, and total bilirubin using the diazo reaction. Glucose concentrations were determined by the glucose oxidase method. Globulin and the A/G ratio were calculated from measured albumin and total protein values.

### 2.4 Genotoxicity assays

#### 2.4.1 Bacterial reverse mutation assay

##### 2.4.1.1 Bacteria strains

Histidine-requiring *S. typhimurium* (TA 100, TA 1535, TA 98, and TA 1537) and tryptophan-requiring *E. coli* (WP2uvrA) strains were used in this study. *S. Typhimurium* strains TA 98 and TA 1537 were used for frame-shift mutagenesis, whereas strains *S. Typhimurium* TA 100 and TA 1535 and *E. coli* (WP2uvrA) were applied for base-pair substitution mutagenesis, both with and without metabolic activation. Test strains were stored as frozen working stock cultures (0.09 mL of dimethyl sulfoxide (DMSO)/ml broth culture) at −80 °C.

##### 2.4.1.2 Mutagenicity assay

In the absence of metabolic activation, the positive controls used were sodium azide (NaN3) for the TA100 and TA1535 strains, furylfuramide (AF-2) for the WP2uvrA strain, 2-Nitrofluorene (2-NF) for the TA98 strain, and acridine mutagen (ICR-191) for the TA1537 strain. When metabolic activation was present, 2-Aminoanthracene (2AA) served as the positive control for all strains. The extract was considered mutagenic if it produced more than a two-fold increase in revertant colonies compared to the negative control, or if it showed a dose-dependent increase in revertant colonies. The positive control mutagens were as follows: acridine mutagen ICR-191, 2-Nitrofluorene, 2-Aminoanthracene, furylfuramide and sodium azide (NaN3) which was dissolved in distilled water, while furylfuramide (AF-2), acridine mutagen (ICR-191), 2-Nitrofluorene (2-NF), and 2-Aminoanthracene (2AA) were dissolved in dimethyl sulfoxide. The mutagenic potential of *D. hispida* aqueous extract was assessed following OECD testing guideline 471 for the bacterial reverse mutation test. In the absence of metabolic activation, the positive controls used were sodium azide (NaN3) for the TA100 and TA1535 strains, furylfuramide (AF-2) for the WP2uvrA strain, 2-Nitrofluorene (2-NF) for the TA98 strain, and acridine mutagen (ICR-191) for the TA1537 strain. When metabolic activation was present, 2-Aminoanthracene (2AA) served as the positive control for all strains. The extract was considered mutagenic if it produced more than a two-fold increase in revertant colonies compared to the negative control, or if it showed a dose-dependent increase in revertant colonies. Triplicate mutagenicity assays were conducted with and without metabolic activation, using a lyophilized rat liver S9 mixture and employing the standard pre-incubation method. For each test, 2,000 mL of overlay agar was added to culture tubes containing 100 µL of bacterial culture (approximately 1 × 10^9^ cells/mL), 100 µL of *D. hispida* aqueous extract at concentrations of 0, 313, 625, 1,250, 2,500, and 5,000 µg/plate, or the appropriate controls-positive mutagens (positive control) or sterile water (negative control) along with 500 µL of the S9 mixture or 500 µL of 0.1 M sodium phosphate buffer (without S9). The concentrations were selected based on preliminary dose-finding tests. The mixtures were vortexed, poured onto the surface of minimal agar plates, and swirled to create an even layer before being allowed to solidify. After incubation at 37 °C ± 2 °C for 48 h, the His + revertant colonies were counted and compared to the negative and positive control plates.

#### 2.4.2 *In vitro* micronucleus assay

V79B cells, a fibroblast-like cells which originated from the Chinese hamster lung, were obtained from the Cell Bank at RIKEN Bio-Resource Center, Japan (Resource no: RBRC-RCB2337). The cells were cultured in Dulbecco’s Modified Eagle’s Medium, supplemented with 10% heat-inactivated fetal bovine serum and 1% v/v antibiotic/antimycotic solution. The cultures were maintained in a humidified CO_2_ incubator with approximately 5% CO_2_ at 37 °C until they reached sub-confluency. Then, the cell growth inhibition of *D. hispida* aqueous extract was tested on V79B. The cytotoxic effects of the extract at concentrations of 5, 2.5, 1.25, 0.625, and 0.3125 mg/mL were evaluated during the dose-finding test. Upon completion of 21 h ± 30 min incubation, the cells were counted, and the cytotoxicity was determined using the formula of Relative Increase in Cell Count (RICC) as below:

$$\text{RICC}=\frac{\text{Increase}\;\text{in}\;\text{number}\;\text{of}\;\text{cells}\;\text{in}\;\text{treated}\;\text{cultures}\;\left(\text{final}\;\text{count}-\text{starting}\;\text{count}\right)}{\text{Increase}\;\text{in}\;\text{number}\;\text{of}\;\text{cells}\;\text{in}\;\text{negative}\;\text{control}\;\text{cultures}\;\left(\text{final}\;\text{count}-\text{starting}\;\text{count}\right)}\times \;100$$

The micronucleus assay was conducted following OECD Testing Guideline 487 for *In Vitro* Mammalian Cell Micronucleus with minor modifications. A total of 1 × 10^5^ cells were cultured for 24 h in a 6-well plate at 37 °C and 5% CO_2_ to allow cell recovery, attachment, and progression into the exponential growth phase. After incubation and confirmation of sub-confluency and morphology, the cells were treated with varying concentrations of *D. hispida* aqueous extract, along with positive and negative controls, both in the presence (+S9) and absence (−S9) of metabolic activation. The cells were exposed to treatments for 3 h, and after treatment, each well was visually inspected for any extract precipitation. The media was discarded, and the cells were carefully rinsed with PBS to preserve cells in mitosis. The plates were incubated at 37 °C ± 2 °C and 5% CO_2_ for 21 ± 0.5 h. After incubation, 2 mL of PBS-EDTA was added, and the cells were resuspended to form a single-cell suspension, followed by centrifugation at 1,500 rpm. Next, the cell pellets were resuspended in 2 mL of potassium chloride (KCl) hypotonic solution and centrifuged again at 1,500 rpm for 5 min. The resulting cell pellet was fixed using Carnoy’s fixative solution, and 10 µL of cells were dropped and mounted onto pre-warmed frosted slides. The cells were stained with 10 µL of 20 µg/mL acridine orange and observed under a fluorescence microscope at 20–40× magnification. Images of 2,000 cells were captured, and at least 1,000 cells were scored for each treatment condition. Non-micronucleated cells (x) and micronucleated cells (y) were analyzed for each treatment group. The frequency of micronucleated cells was expressed as a percentage using the formula:

$$\%\text{ Micronucleated Cells}=\frac{y}{y+x}\;\times \;100$$

The mean of the percentage of micronucleated cells was then calculated.

### 2.5 Statistical analysis

All data collected during the study has been recorded and analyzed. The mean value (x), standard deviation (SD), and standard error of the mean (SEM) were calculated for each variable measured using Microsoft Office Excel version 2,403 (Analysis ToolPak). Statistical analysis was not performed for the acute toxicity study as the fixed dose procedure uses predefined doses (5, 50, 300, and 2,000 mg/kg) with a small number of animals, aiming to identify a dose that produces evident signs of toxicity without causing death, resulting in categorical rather than quantitative outcomes such as lethality dose at fifty percent population (LD_50_). Statistical comparisons between treated groups and the negative control for *in vitro* micronucleus and bacterial reverse mutation assay were conducted using one-way ANOVA, followed by Dunnett’s multiple comparison test. A *p*-value of <0.05 was considered statistically significant.

## 3 Results

### 3.1 Qualitative phytochemistry

The phytochemistry test revealed that *D. hispida* aqueous extract contains alkaloid, saponin, and flavonoid while tannin, polyphenol, triterpene, and steroids were not detected (Table 1).

**TABLE 1:** Qualitative phytochemistry test.

Constituents	Results
Alkaloids	2+
Saponins	1+
Flavonoids	2+
Tannins/Polyphenol	-
Triterpenes/Steroids	-

### 3.2 Acute toxicity study

During the 14-day observation period, no significant behavioural changes were noted in any of the animals. There was no treatment-related mortality, and the internal organs of all rats displayed no clinical signs of toxicity, even at the highest dose of 2,000 mg/kg body weight. Compared to the sighting groups, the rats in the main study group exhibited slightly lower weight gain, but no abnormal fluctuations in body weight were observed across the experimental groups (Table 2). Additionally, no differences were noted in food and water intake between the sighting and main study groups.

**TABLE 2:** Effects of oral administration of *Dioscorea hispida* aqueous extract on acute lethal effect, water and feed intake, and body weight gain (%) in rats.

Groups	Dose (mg/kg BW)	No. of death after 24 h	Water intake (mL)	Feed intake (gram)	Weight gain (%)
Sighting (n = 1)	5	0	610	269.8	8.2
	50	0	353	222.9	6.6
	300	0	800	264.7	18.3
Main Study (n = 5)	2000	0/5	496 ± 88.8	224.3 ± 13.7	5.6 ± 0.7

#### 3.2.1 Clinical observation

Throughout the study, no signs of toxicity were detected. There were no changes observed in the skin, fur, eyes, or mucous membranes. The rats exhibited normal function in the respiratory, circulatory, autonomic, and central nervous systems. Additionally, there were no abnormalities in somatomotor activity or behavioral patterns. None of the rats showed symptoms such as tremors, convulsions, excessive salivation, diarrhea, lethargy, sleep disturbances, or coma.

#### 3.2.2 Organs weight

During the gross examination, no abnormalities were observed in any of the organs. The organ-to-body weight ratios of all rats, as presented in Table 3, showed no significant differences. In the *D. hispida* aqueous extract main group, the ratios were comparable to those in the sighting groups.

**TABLE 3:** Effects of oral administration of *Dioscorea hispida* aqueous extract on organ-body weight ratios (%) in rats.

(%) Organ-body weight ratio				
Groups	Sighting			Main study
Organ	5 mg/kg BW (n = 1)	50 mg/kg BW (n = 1)	300 mg/kg BW (n = 1)	2000 mg/kg BW (n = 5)
Lung	1.593	1.06	1.215	1.326 ± 0.105
Heart	0.915	0.785	0.828	0.85 ± 0.051
Spleen	0.471	0.404	0.501	0.438 ± 0.051
Stomach	1.397	1.232	1.636	1.314 ± 0.151
GIT	6.940	6.189	7.321	6.565 ± 1.579
Liver	6.973	5.579	10.616	6.276 ± 0.855
Kidney (R)	0.743	0.716	0.856	0.73 ± 0.033
Kidney (L)	0.793	0.74	0.817	0.767 ± 0.015
Adrenal (R)	0.034	0.022	0.036	0.028 ± 0.005
Adrenal (L)	0.030	0.026	0.031	0.028 ± 0.00
Ovary (R)	0.089	0.060	0.080	0.075 ± 0.014
Ovary (L)	0.074	0.054	0.098	0.064 ± 0.013

#### 3.2.3 Biochemical analysis

Table 4 presents the effects of oral administration of *D. hispida* aqueous extract on the biochemical parameters in rats. Several biochemical markers, particularly liver function indicators such as AST, ALT, ALP, total protein, and globulin, showed some variations. These marker levels were lower in the main group compared to the sighting groups. Additionally, calcium, glucose, and cholesterol levels were also lower. However, these variations were not found to be dose-related.

**TABLE 4:** Effects of oral administration of *Dioscorea hispida* aqueous extract on biochemical parameters in rats.

Groups/Biochemical parameters	Sighting			Main study
	5 mg/kg BW (n = 1)	50 mg/kg BW (n = 1)	300 mg/kg BW (n = 1)	2000 mg/kg BW (n = 5)
Albumin (g/L)	16	15	19	14.33 ± 1.15
Albumin/Globulin Ratio	0.2	0.2	0.3	0.23 ± 0.06
ALP (U/L)	158	104	158	76 ± 17.35
ALT (U/L)	101	106	81	69.33 ± 13.05
Amylase (U/L)	456	601	682	475.33 ± 4.62
AST (U/L)	222	286	202	178 ± 1.5
BUN (mmol/L)	5.6	6	8.2	6.53 ± 0.21
Calcium (mmol/L)	3.21	3.34	3.11	2.54 ± 0.16
Cholesterol (mmol/L)	3.5	3.2	3.2	2.27 ± 0.21
Glucose (mmol/L)	12.5	13.65	13.88	9.19 ± 0.72
Total Bilirubin	5	6	3	3.67 ± 1.15
Total Protein	107	78	78	67.67 ± 6.42
Globulin	91	63.9	59	53.33 ± 6.11

ALP, Alkaline phosphatase; ALT, Alanine transaminase; AST, Aspartate transaminase; BUN, blood urea nitrogen.

### 3.3 Bacterial reverse mutation test

The results of the bacterial reverse mutation assay were summarized in Tables 5, 6. At concentrations of 313, 625, 1,250, 2,500, and 5,000 µg/plate, there were no significant increases in the number of revertant colonies for any test strain (TA100, TA1535, TA98, TA1537, and WP2uvrA), either with or without the S9 mixture, when compared to the negative control group. Additionally, no bacterial toxicity was observed in any of the test strains at the doses tested. The mean number of revertant colonies in the positive control group was consistently higher than in the negative control group for all strains.

**TABLE 5:** Results of bacterial reverse mutation assay of base-pair substitution type of *Dioscorea hispida* aqueous extract on *S. Typhimurium* TA 100, TA 1535, and *Escherichia coli* WP2uvrA with (+S9) and without (−S9) metabolic activation.

Strains	Number of revertant colonies [(number of colonies/plate) ± SD]					
	TA100		TA1535		WP2*uvr*A	
Concentration (µg/plate)	(+S9)	(−S9)	(+S9)	(−S9)	(+S9)	(−S9)
313	149 ± 8	167 ± 7	14 ± 1	18 ± 2	43 ± 3	51 ± 4
625	150 ± 6	166 ± 3	14 ± 1	15 ± 1	49 ± 6	49 ± 3
1,250	148 ± 6	162 ± 8	16 ± 1	17 ± 3	43 ± 2	51 ± 4
2,500	157 ± 3	165 ± 5	15 ± 1	17 ± 2	45 ± 5	45 ± 7
5,000	143 ± 5	150 ± 8	17 ± 2	18 ± 2	59 ± 3	44 ± 6
Negative Control (Water)	156 ± 4	156 ± 2	18 ± 1	19 ± 1	42 ± 3	44 ± 3
Positive control	NaN_3_	2AA	NaN_3_	2AA	AF-2	2AA
Concentration (µg/plate)	0.5	1.0	0.5	2.0	0.05	10.0
Revertant	^a^817 ± 5	^a^626 ± 7	^a^557 ± 9	^a^244 ± 12	^a^772 ± 8	^a^133 ± 5

NaN_3_ - sodium azide; 2-AA - 2-aminoanthracene; AF-2, Furyl furamide. Data were expressed as mean ± SD.

^a^ significant increase when compared to treatment and negative groups (*p* < 0.05).

**TABLE 6:** Results of bacterial reverse mutation assay of frame-shift type of *Dioscorea hispida* aqueous extract on *S. Typhimurium* TA 98 and TA 1537 with (+S9) and without (−S9) metabolic activation.

Strain	Number of revertant colonies [(number of colonies/plate) ± SD]			
	TA 98		TA 1537	
Concentration (µg/plate)	(+S9)	(−S9)	(+S9)	(−S9)
313	31 ± 4	21 ± 7	9 ± 2	11 ± 2
625	31 ± 3	23 ± 3	11 ± 2	12 ± 2
1,250	32 ± 4	24 ± 8	9 ± 2	10 ± 2
2,500	31 ± 4	24 ± 5	12 ± 2	10 ± 1
5,000	38 ± 3	25 ± 2	12 ± 2	10 ± 2
Negative Control (Water)	32 ± 4	28 ± 2	9 ± 1	11 ± 1
Positive control	2AA	2-NF	2AA	ICR-191
Concentration (µg/plate)	0.5	1.0	0.5	2.0
Number of revertant	^a^149 ± 15	^a^514 ± 34	^a^159 ± 8	^a^748 ± 4

2NF, 2-nitrofluorene; 2-AA - 2-aminoanthracene; ICR-191, Acridine mutagen. Data were expressed as mean ± SD.

^a^ significant increase when compared to treatment and negative groups (*p* < 0.05).

### 3.4 *In vitro* micronucleus test

No cytotoxicity was observed in the treatment dose-finding test. As a result, the three highest concentrations were selected for the micronucleus assay. There were no significant or dose-dependent increases in the percentage of micronucleated cells at any *D. hispida* aqueous extract dose level compared to the negative control group, regardless of the presence or absence of metabolic activation (Table 7). In contrast, the positive control showed the highest percentage of micronucleated cells under both conditions, with and without metabolic activation.

**TABLE 7:** Effects of 3-h treatment of *Dioscorea hispida* aqueous extract or positive control with (a) and without (b) the presence of S9 metabolic activation on V79B cells in the micronucleus assay.

Groups	RICC		Percentage of micro-nucleated cells	
	−S9	+S9	−S9	+S9
Negative Control	100.0	100.0	0.2	0.9
Positive Control	100.0 (Colchicine)	44.0 (cyclophosphamide)	3.2	11.1
DHAE 1.25 mg/mL	66.0	76.5	0.2	0.7
DHAE 2.5 mg/mL	84.0	87.5	0.3	0.8
DHAE 5 mg/mL	47.0	75.0	0.2	0.8

*RICC, Relative Increase in Cell Counts.

## 4 Discussion

Research on the therapeutic properties of herbal plants has been increasing globally as more people turn to traditional and alternative complementary medicine. *Dioscorea* spp. have long been consumed and traditionally prepared in powder form as a source of carbohydrates. Scientifically, the tuber of *Dioscorea* has demonstrated various pharmacological effects, including antioxidant, anti-tumor, anti-inflammatory, cholesterol-lowering properties, diabetes, syphilis, and menstrual disorders (Kundu et al., 2021; Chaniad et al., 2020). Chemical compounds in herbal plants, such as alkaloids, flavonoids, and terpenoids, play key roles in therapeutic efficacy by interacting with specific biological targets to produce desired pharmacological effects. The qualitative phytochemistry assay conducted in the present study confirmed the presence of alkaloids, saponin and flavonoids in the *D. hispida* aqueous extract. While these bioactive compounds offer promising therapeutic potential, their presence also raises safety concerns if consumed in high doses, for prolonged periods, or due to bioaccumulation or interactions with other substances that warrant careful toxicological evaluation.

Subchronic exposure to high doses (300 mg/kg body weight) of *D. hispida* ethanol extract over 90 days has been shown to cause significant hepatic and renal damage, as indicated by elevated levels of creatinine, ALT, and AST, alongside histopathological changes (Syahputra et al., 2025). Moreover, Muhammad et al. (2022) reported that oral administration of *D. hispida* aqueous extract at 250 and 500 mg/kg resulted in oxidative stress and DNA damage in placental tissues of rats (Muhammad et al., 2022). These findings highlight the necessity of conducting thorough pre-clinical toxicity evaluations to ensure the safety of *D. hispida*-based products before advancing to clinical trials.

In herbal product development, an acute toxicity study is conducted to determine its safe dosage range, identify immediate toxic effects, comply with regulatory requirements, and ensure the extract is non-lethal before progressing to further toxicological or pharmacological evaluations. The present study showed that a single dose of *D. hispida* aqueous extract up to 2,000 mg/kg body weight did not result in mortality or behavioral changes in the treated animals. Similar findings have been reported in studies using various extracts of *Dioscorea* species. According to Lima et al. (2013) the administration of 5,000 mg/kg aqueous extract of *Dioscorea villosa* did not cause mortality, weight loss, or toxicity in Wistar rats (Lima et al., 2013). Similarly, no adverse effects were observed with the single dose administration of *Dioscorea oppositifolia* methanol extract at 2,000 mg/kg body weight (Jhansirani et al., 2010). Thus, the results indicated that *D. hispida* aqueous extract was not toxic at a single dose up to 2,000 mg/kg body weight. Further studies on repeated dose are required to evaluate the adverse effects of a *D. hispida* aqueous extract following prolonged exposure, determine target organ toxicity, establish the no-observed-adverse-effect level (NOAEL), and support the safety assessment for its long-term use.

Despite differences in some clinical biochemistry values among female rats in the acute toxicity study of *D. hispida* aqueous extract between the sighting and main study groups, no clinical significance can be concluded. Levels of liver enzymes like ALT, AST and ALP are commonly used to assess hepatocellular injury. In the main study group 2,000 mg/kg, the mean liver enzymes are lower than or comparable to those seen in the sighting groups, suggesting no dose-dependent increase and no hepatocellular damage at the highest tested dose. These enzyme levels fall within acceptable physiological ranges for rodents and indicate preserved liver integrity. Similarly, kidney function markers such as BUN remained within physiologically acceptable limits, indicating preserved renal function. These results are consistent with previous studies on the acute toxicity of *Dioscorea deltoidea* cell biomass where no significant changes on such parameters at 2,000 and 5,000 mg/kg (Titova et al., 2021). Likewise, Lima et al. (2013) demonstrated that *D. villosa* aqueous extract at 5,000 mg/kg did not produce significant changes in biochemical markers or cause mortality in Wistar rats, aligning with the safety profile observed in this study. Total protein, albumin, and globulin levels in the main study group were slightly lower compared to the sighting phase but remained within normal physiological limits. This reduction could be attributed to biological variability rather than a treatment-related effect, especially considering the absence of clinical signs or histopathological lesions. Furthermore, the lack of adverse biochemical changes in this study supports the assertion that acute exposure to the aqueous extract is non-toxic, although caution is warranted when extrapolating these results to long-term use.

Genotoxicity testing is essential for identifying potential genotoxic carcinogens or mutagens. It plays a key role in the safety assessment of herbal medicines, particularly their effects on genetic material, which can lead to cell mutations (Saks et al., 2017). To evaluate the genotoxic potential of *D. hispida* aqueous extract, two assays were conducted, including the bacterial reverse mutation (Ames) test and an *in vitro* micronucleus test. The Ames test is a primary screening method for detecting genotoxicity or mutagenicity of herbal substances using *Salmonella* and *E. coli* bacterial strains (Lorin et al., 2013; Hwang et al., 2012). In this study, *D. hispida* aqueous extract was found to be non-mutagenic in all tested strains, with no significant increase in the number of revertant colonies, even in the presence of metabolic activation at all concentrations used. Conversely, the positive controls (2-AA, NaN3, MMC, 2-NF, and ICR-191) significantly induced mutation frequencies, as shown by the increased number of revertant colonies in all strains. This lack of mutagenicity of *D. hispida* aqueous extract may be attributed to the presence of antioxidants and phytochemicals in this herbal plant (Jeong et al., 2018). It has been noted that *D. hispida* exhibits antioxidant activity, and phytochemical analyses have identified antioxidant compounds such as phenols and flavonoids (Adomėnienė and Venskutonis, 2022; Obidiegwu et al., 2020). These phenolic compounds and flavonoids are recognized for their ability to mitigate the genotoxicity of certain carcinogenic substances through their radical scavenging properties (Prakash et al., 2014). Likewise, numerous studies have reported the antioxidant and antimutagenic properties of various *Dioscorea* species (Kumar et al., 2017). These results align with the findings of Cha et al., 2021 who assessed the genotoxic potential of *Dioscorea rhizome* water extract reporting no mutagenic response even at the highest concentration (5,000 µg/plate) (Cha et al., 2021). In contrast, a study conducted by Yoon (2013) demonstrated that *Dioscorea batatas* exhibited mutagenic effects on *Salmonella* tester strains when extracted with 70% ethanol and in the presence of metabolic activation (Yoon, 2013). These findings suggests that the mutagenicity may vary based on the extraction solvent used and require further investigations (Cha et al., 2021; Lorin et al., 2013).

The *in vitro* micronucleus assay is a standard genotoxicity test that detects chromosome damage in cultured cells and is used to evaluate the potential of a substance, including herbal products, to cause genetic mutations. In the *in vitro* micronucleus assay, *D. hispida* aqueous extract did not induce any significant increase in the number of micronuclei in any tested dose of *D. hispida* aqueous extract compared with the negative control. These results are also consistent with the study of the potential genotoxic effect of *D. rhizome* water extract on ICR mice using a bone marrow micronucleus assay. These results have shown that *D. hispida* aqueous extract was not mutagenic in the absence and presence of metabolic activation towards V79B cells (Cha et al., 2021).

## 5 Conclusion

*D. hispida* aqueous extract demonstrated no acute toxicity or genotoxic effects in both *in vivo* and *in vitro* models. The extract was well tolerated up to 2,000 mg/kg body weight in rats and showed no mutagenic or clastogenic activity in bacterial reverse mutation and micronucleus assays. These findings suggest that *D. hispida* aqueous extract is safe for short-term use, although further preclinical studies are necessary to evaluate its safety under repeated dosing and *in vivo* genotoxicity conditions.

## Data Availability

The original contributions presented in the study are included in the article/supplementary material, further inquiries can be directed to the corresponding author.
